# Mitochondrial function influences expression of methamphetamine-induced behavioral sensitization

**DOI:** 10.1038/s41598-021-04301-9

**Published:** 2021-12-31

**Authors:** I. Daphne Calma, Amanda L. Persons, T. Celeste Napier

**Affiliations:** 1 Departments of Psychiatry and Behavioral Sciences, Rush University Medical Center, Chicago, IL 60612 USA; 2 Departments of Physician Assistant Studies, Rush University Medical Center, Chicago, IL 60612 USA; 3 Center for Compulsive Behavior and Addiction, Rush University Medical Center, Chicago, IL 60612 USA; 4 Department of Psychiatry and Behavioral Sciences, Rush University Medical Center, Suite 424, Cohn Research Building, 1735 W. Harrison Street, Chicago, IL 60610 USA

**Keywords:** Diseases of the nervous system, Neuroscience, Diseases, Neurology

## Abstract

Repeated methamphetamine use leads to long lasting brain and behavioral changes in humans and laboratory rats. These changes have high energy requirements, implicating a role for mitochondria. We explored whether mitochondrial function underpins behaviors that occur in rats months after stopping methamphetamine self-administration. Accordingly, rats self-administered intravenous methamphetamine for 3 h/day for 14 days. The mitochondrial toxin rotenone was administered as (1 mg/kg/day for 6 days) via an osmotic minipump starting at 0, 14 or 28 days of abstinence abstinence. On abstinence day 61, expression of methamphetamine-induced behavioral sensitization was obtained with an acute methamphetamine challenge in rotenone-free rats. Rotenone impeded the expression of sensitization, with the most robust effects obtained with later abstinence exposure. These findings verified that self-titration of moderate methamphetamine doses results in behavioral (and thus brain) changes that can be revealed months after exposure termination, and that the meth-initiated processes progressed during abstinence so that longer abstinence periods were more susceptible to the consequences of exposure to a mitochondrial toxin.

## Introduction

Methamphetamine (meth) is a highly abused psychomotor stimulant. Meth has a complex pharmacology with direct and indirect consequences that differ depending on the dosing protocols used. Moderate, non-contingent bolus doses of meth given in vivo (e.g., 2.5–10 mg/kg) increase neuronal cytosolic, synaptic and extra-synaptic levels of dopamine, norepinephrine, and serotonin^1,2^. The increase in transmission can lead to neuroplasticity that involves profound and persistent biochemical and structural changes in neuronal elements, including cytoskeleton reorganization, synthesis and translocation of key proteins, influx of calcium, and activation of kinases^3–8^. Such neuroplastic events increase neuronal energy demands. Mitochondria are the primary source of cellular energy (i.e., ATP), and mitochondrial dysfunction leads to long term or permanent damage in the brain^9–12^. Such dynamic events can continue long after drug exposure has ended^13–17^, implicating a role for mitochondria during processes associated with abstinence from chronic exposure to meth. For example, non-contingently administered meth (7.5–10 mg/kg to rats) reduces mitochondrial respiration and inhibits complex II of the electron transport chain when meth is no longer in the system^18,19^, but the persistence of the mitochondrial-induced effects is unclear. To interrogate this unknown, the current study evaluated the ability of a systemically available mitochondrial toxin, rotenone, to alter meth-induced behaviors, when the rats were tested weeks to months after toxin exposure.

The repeated use of meth can lead to the development of addiction. Addiction reflects maladaptive plasticity of neuronal systems that are engaged during drug-associated learning^7,20–25^ and persist long after drug-taking ceases^26–28^. In laboratory animals, behavioral manifestations of meth-induced plasticity include sensitization^29–32^. Behavioral sensitization is used to describe enhancements in motor responding to bolus administration of moderately high doses of abused drugs wherein the experimenter determines treatment dose (i.e., non-contingent administration)^31,33–38^, e.g., 5–20 mg/kg of drug given once daily for 5 days^29–31,39,40^. Expression of behavioral sensitization is often measured following an acute challenge of the stimulant, administered after a period of abstinence^29–31,41,42^. The hypothetical construct for this model is that the adaptive processes in brain circuits that govern drug-induced motor function parallel those that govern reward-motivated behavior; therefore, motor readouts from drug-treated rats model the adaptive processes that occur in the brains of humans that abuse drugs^41,43^.

We previously determined that behavioral sensitization in laboratory rats develops during repeated exposure to non-contengently administered meth^13,30^, and that this effect persists for up to 60 days, wherein expression intensity directly correlates with abstinence duration^30,44^. The current study was designed to interrogate the long-term involvement of mitochondria in meth-induced behavioral sensitization using the mitochondrial toxin rotenone. We posed that if sensitization is a dynamic, mitochondrial-dependent process, then the effects of rotenone would differ depending on the time of rotenone exposure after terminating meth self-administration.

## Methods

### Animals

Male Sprague–Dawley rats (n = 104; Envigo, Indianapolis, IN) weighing 225–250 g upon arrival were housed in pairs, handled daily, and acclimated to environmentally-controlled conditions (temperature set point 22 °C, humidity set 40–45%) for at least one week prior to the start of the experimental protocols. Rats had access food and water *ad libtum*. All procedures were performed in accordance with the Guide for the Care and Use of Laboratory Animals (National Research Council, Washington DC) with protocols approved by the Rush University Institutional Animal Care and Use Committee and with the Animal Research: Reporting of In Vivo Experiments (ARRIVE) guidelines.

### Surgical procedures

Jugular vein catheter implantation followed our published protocols^45–47^. Rats were deeply anesthetized with 2–3% isoflurane. Catheters constructed of silastic tubing (0.3 mm × 0.64 mm; Dow Corning Co., Midland, MI) were inserted into the right jugular vein and secured with sutures. The distal end of the tubing passed subcutaneously over the mid-scapular region and exited through a metal guide cannula (22 gauge; Plastics One Inc., Roanoke, VA). Rats recovered for one week during which the catheters were flushed daily with 0.1–0.2 mL sterile saline to maintain patency.

**Figure 1:**
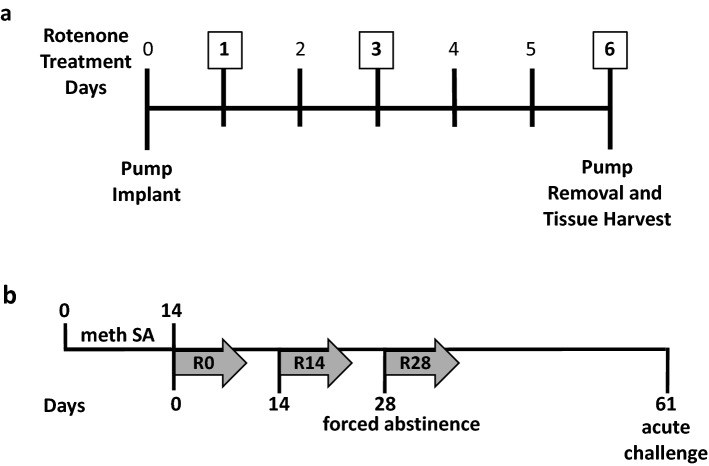
Study timelines. (**a**) Alzet® minipumps set to deliver vehicle, 1 mg/kg/day or 3 mg/kg/day of rotenone were implanted subcutaneously. Behavioral measures were conducted on days 1, 3 and 6 of treatment. Pumps were then removed and tissues were harvested at the end of treatment day 6. (**b**) Rats self-administered meth 3 h/day for 14 days via lever pressing or were non-contingently given meth via subcutaneous injection. Alzet® minipumps containing vehicle or 1 mg/kg/day of rotenone were implanted subcutaneously on meth forced abstinence day 0, 14, or 28. Pumps were then removed and tissues were harvested at the end of treatment day 6. Rats abstained from all treatment until forced abstinence day 61, which is when all rats were subcutaneously administered 1 mg/kg meth (meth acute challenge) and behavioral measures were collected. On forced abstinence day 62, rats were sacrificed and tissues were collected.

For implantation of osmotic minipumps, rats were anaesthetized with 2–3% isoflurane. The pumps (Model 2001, Alzet, Cupertino, CA) were inserted subcutaneous (sc) between the shoulder blades for the rotenone dose–response pilot study (Fig. 1a), and above the left hind limb for rats tested during meth abstinence (Fig. 1b). For both studies, the pumps were removed after six days under isoflurane anesthesia.

### Drugs and treatment protocols

Rotenone (Sigma-Aldrich, St. Louis, MO) was dissolved in a vehicle solution of 1:1 mixture of dimethyl sulfoxide (DMSO; Sigma-Aldrich) and polyethylene glycol (PEG; Sigma-Aldrich) and was administered via sc implanted minipumps. Guided by prior studies^48–51^, a pilot dose–response study evaluated motor responses after rotenone administered as 1 mg/kg/day (n = 4) or 3 mg/kg/day (n = 4) *versus* vehicle (n = 4) for six days. Several studies demonstrate that chronic systemic rotenone treatments with 2–3 mg/kg/day for 6–10 days, result in mitochondrial dysfunction and motor deficits^47–49,52^. The objectives of the current pilot study were to validate a dosing protocol that was sufficient to alter motor function, and to identify a protocol that was subthreshold to this effect, i.e., one that did not alter motor function. Motor assessments were conducted on the first, third and last day of the rotenone treatment (Fig. 1a). To do so, rats were transported from the housing area to the test area 30 min prior to being placed in automated motor activity test boxes for a 60 min test session. As previously published with these boxes^17,53–56^, the rats were the most active for the first 15 min, and data from this time period were used to define ‘threshold’ and ‘subthreshold’ doses of rotenone.

(+) Methamphetamine HCl (Sigma-Aldrich, St. Louis, MO) was dissolved in sterile saline. *Self-administration:* Meth was administered intravenously (iv) as 0.1 mg/kg per 0.1 mL infusion. Self-administration sessions were 3 h per day for a total of 14 days (Fig. 1b). Rats self-administered meth on a fixed ratio-1 (FR-1) schedule of reinforcement wherein one press on the active lever resulted in an infusion of meth and illumination of the cue light for 6 s; the house light illuminated for 20 s indicating a time-out period. Presses on the inactive lever had no programmed consequences. Control rats were yoked to a meth counterpart, so that each infusion of meth resulted in a 0.1 mL infusion of saline vehicle. Lever presses by saline-yoked rats had no programmed consequences. Number of active lever presses, inactive lever presses, and the number of infusions were recorded. *Repeated non-contingent administration of meth:* Rats that lost catheter patency within the first 5 days of self-adminstration were moved to a non-contingent group to serve as controls for the operant task. These rats were “matched” with a meth self-administering rat, and they received a sc bolus injection that equaled the daily intake of their self-administering counterpart. *Acute meth challenge:* On forced abstinence day 61, rats were transported from the housing area to the test area 30 min prior the start of motor assessments. Rats were placed in the motor boxes (AccuScan Instruments, Inc., Columbus, OH) for 30 min, then returned to their home cage, non-contingently administered 1 mg (base)/kg sc injection of meth (referred to as an acute challenge) and immediately placed back into the motor boxes. Behavioral responses to the acute challenge was measured for 90 min (described in Table 1). The motor boxes were equipped with two banks of infrared photocells, which allowed for quantification of motor function in three dimensional space. The data were tallied for two test session times i.e., the onset and peak of meth-induced effects. Data for onset were summed for the 20–30 min period after the meth injection, illustrating when meth begins to act in the brain. Data for the peak effect were summed for 30–50 min post injection, illustrating when meth concentrations are likely the highest in the brain^57^.

**Table 1:** Descriptions of parameters utilized for motoric measures.

Motor measure	Description
Horizontal beam breaks	The number of interruptions of the horizontal sensors
Total distance	The horizontal distanced traveled measured by the interruptions of the horizontal sensors reported in centimeters
Vertical beam breaks	The number of beam interruptions that occurred in the vertical sensor
Vertical movement number	The number of occurrences the rat spends at least 1 s between two successive beam interruptions of the vertical sensors
Vertical time	The time between two successive beam interruptions of the vertical sensors
Stereotypy count	The number of occurrences when the rat interrupts the same sensors repeatedly
Stereotypy number	The number of occurrences when there is at least 1 s between successive repetitive interruptions of the same sensor
Stereotypy time	The time between successive repetitive interruptions of the same sensor

At the end of the operant task, rats were randomly assigned to receive a sc minipump containing 1 mg/kg/day of rotenone or vehicle for six days during forced abstinence. A 2 × 2 factorial design was used: saline/vehicle, saline/rotenone, meth/vehicle, and meth/rotenone. The study was conducted in two runs, each run containing equal number of rats from the four treatment groups. To determine if the abstinence time from meth had an effect on the impact of rotenone treatment, the toxin (or its vehicle) were administered at three different forced abstinence time periods: abstinence day 0 thru day 6 (early, R0), forced abstinence day 14 thru day 20 (mid, R14), or abstinence day 28 thru day 34 (late, R28) (Fig. 1b).

### Statistical analyses

For the rotenone dose–response study, a one-way ANOVA with a post hoc Dunnett’s test was conducted to detect differences between vehicle and each dose of rotenone (1 mg/kg or 3 mg/kg/day) within days 1, 3 and 6 of treatment. For the self-administration study, a two-way repeated measures ANOVA was used to determine the difference between active and inactive lever presses across self-administration days followed by a post hoc Bonferonni’s test to detect differences within each self-administration day. A one-way ANOVA with a post hoc Newman-Keuls was used determine differences for cumulative meth intake among the R0, R14 and R28 three groups. For motor outcomes, saline/vehicle and meth/vehicle rats were compared using a one-tailed Student’s *t*-test. Rotenone assessments were done using a two-way ANOVA with a post hoc Newman-Keuls. Saline/rotenone vs saline/rotenone comparisons among the three different rotenone administration times (R0, R14, and R28) were compared to determine whether the time of rotenone treatment altered motor activity. Meth/rotenone vs meth/rotenone comparisons among the three different rotenone administration times (R0, R14, and R28) were compared to determine whether rotenone treatment time had an effect on behaviors produced by meth self-administration. Saline/rotenone vs meth/rotenone comparisons within each treatment time frame were conducted to determine whether rotenone altered meth-induced behavioral sensitization.

Contingency comparisons were done with a one-tailed Student’s *t-*test with a Bonferroni adjusted *alpha* of 0.025 to account for multiple comparisons. Data are presented as mean + SEM or mean ± SEM. Statistical analyses were performed using Graphpad Prism software v 8.4.2, (La Jolla, CA).

## Results

### Rotenone dose determination

**Figure 2:**
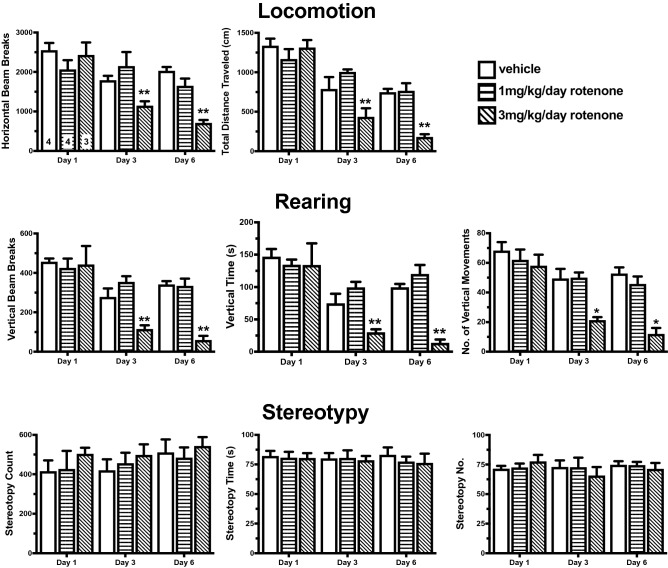
Rotenone dose–response study. Vehicle or rotenone (1 mg/kg/day or 3 mg/kg/day) was administered for 6 days via a subcutaneous osmotic minipump. Assessments of locomotion, rearing and stereotypy were conducted on days 1, 3 and 6. Locomotion and rearing behaviors were significantly decreased in rats treated with 3 mg/kg rotenone on days 3 and 6. There was no effect of rotenone on stereotypy behaviors. One-way ANOVA with a Dunnett’s post hoc test, *p < 0.05, **p < 0.01.

The effects of rotenone were identified using motor function as the outcome (Fig. 2). Rotenone treatments significantly reduced (i) locomotion on day 3 (horizontal activity: F_(2,10)_ = 4.19, *p* < 0.05; total distance: F_(2, 10)_ = 6.20, *p* < 0.05) and day 6 (horizontal activity F_(2, 10)_ = 22.84, *p* < 0.01; total distance F_(2, 10)_ = 21.33, *p* < 0.01), and (ii) rearing on day 3 (vertical activity F_(2, 10)_ = 11.64, *p* < 0.01; vertical movement number, F_(2, 10)_ = 10.56, *p* < 0.01; vertical time F_(2, 10)_ = 11.08, *p* < 0.01) and day 6 (vertical activity F_(2, 10)_ = 29.97, p < 0.01; vertical movement number F_(2, 10)_ = 20.18, *p* < 0.01; vertical time F_(2, 10)_ = 30.01, *p* < 0.01). Rotenone did not alter measures of stereotypy (count F_(2, 10)_ = 0.16, *p* $\ge$ 0.05, number F_(2, 10)_ = 0.78, *p* $\ge$ 0.05, time F_(2, 10)_ = 0.74, *p* $\ge$ 0.05) Post hoc Dunnett’s test reveled that 3 mg/kg/day of rotenone differed from vehicle for locomotion and rearing on days 3 and 6. In contrast, treatment with 1 mg/kg/day rotenone had no effect on any motor outcome assessed. The findings with 3 mg/kg/day for 6 days were consistent with published reports with reduced motor function^48,49^. As 1 mg/kg/day for 6 days did not result in significant motor changes, this treatment was deemed to be ‘subthreshold’, and the protocol was used in subsequent experiments.

### Acquisition of meth self-administration

**Figure 3:**
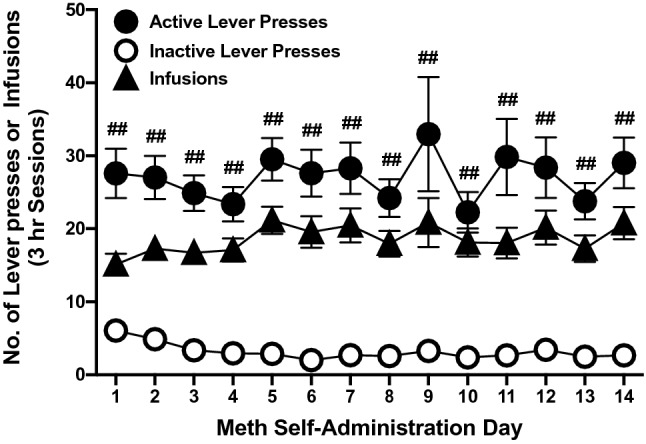
Methamphetamine (meth) self-adminsitration operant task. Meth self-administering rats (n = 42) readily acquired the operant lever pressing task. There was a significant difference between the active and inactive lever presses throughout the operant task. Repeated measures ANOVA followed by a Bonferonni’s post hoc analysis, ^##^p < 0.01 *versus* inactive presses.

All meth self-administering rats readily acquired the operant task (Fig. 3); during the first day of training, selection of the active lever was significantly greater than the inactive lever, indicating that rats were able to differentiate between the reinforced and non-reinforced levers. There was a significant effect of lever presses (F_(1,82)_ = 98.7, *p* < 0.01), but no effect of time (F_(13,1066)_ = 1.1, *p* > 0.05) or a lever press x time interaction (F_(13,1066)_ = 0.9, p > 0.05). Saline-yoked rats exhibited minimal lever pressing on either lever across all sessions (data not shown). The average total (lifetime) intake of meth for all rats tested was 22.0 ± 13.9 mg/kg, with an average daily intake of 1.6 ± 1.0 mg/kg (Table 2). These intakes fall within the calculated rodent equivalency range (1.5–4.0 mg/kg) for doses that humans take during recreational use of meth (0.25–0.67 mg/kg) (Goodman and Gilman 1985). However, there was a significant difference among the three groups for the average total (F_(2, 39)_ = 7.4, *p* < 0.01) and average daily intake (F_(2, 39)_ = 4.9, *p* < 0.05). Post hoc analysis revealed that the R0 treatment group had significantly lower total and daily meth intake compared to R14 and R28 rats (Table 2).

**Table 2:** Methamphetamine (meth) intake following 14 days of self-administration.

mg/kg	R0	R14	R28
Average total meth intake	12.6 ± 1.4	26.1 ± 3.4 **	30.1 ± 5.0 **
Average daily meth intake	1.0 ± 0.1	1.8 ± 0.3 *	2.2 ± 0.4 *

One-way ANOVA with a post hoc Newman-Keuls, *p < 0.05, **p < 0.01 *versus* R0.

### Expression of sensitization following meth acute challenge

**Figure 4:**
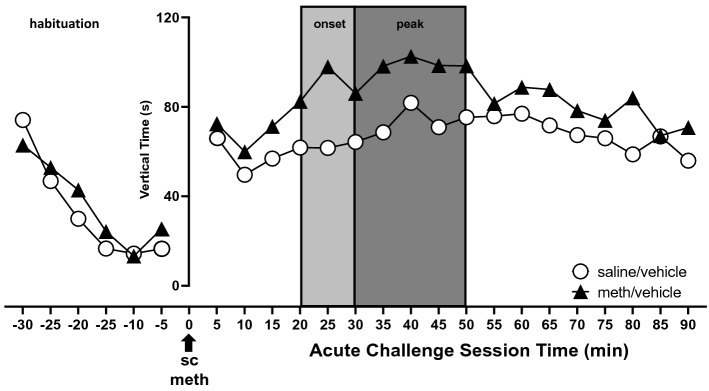
Graphical representation of the response to an acute challenge of methamphetamine (meth). There was no difference in the habituation profile. Shaded boxed indicate the time frames analyzed for onset and peak phases. Error bars were omitted for clarity. Re-label the x-axis as “Time (min) from Meth Acute Challenge”.

**Figure 5:**
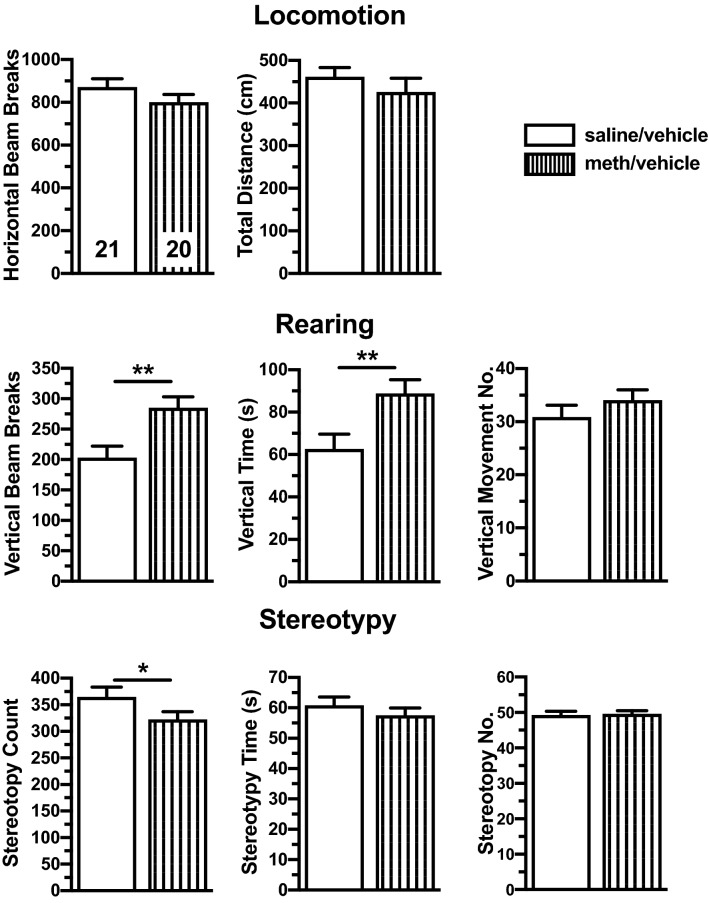
Onset of methamphetamine (meth)-induced behavioral sensitization. Meth/vehicle rats exhibited enhanced vertical outcomes (beam breaks and time) and reduced stereotypy count compared to saline/vehicle rats. One-tailed Student’s t-test, *p < 0.05, **p < 0.01.

**Figure 6:**
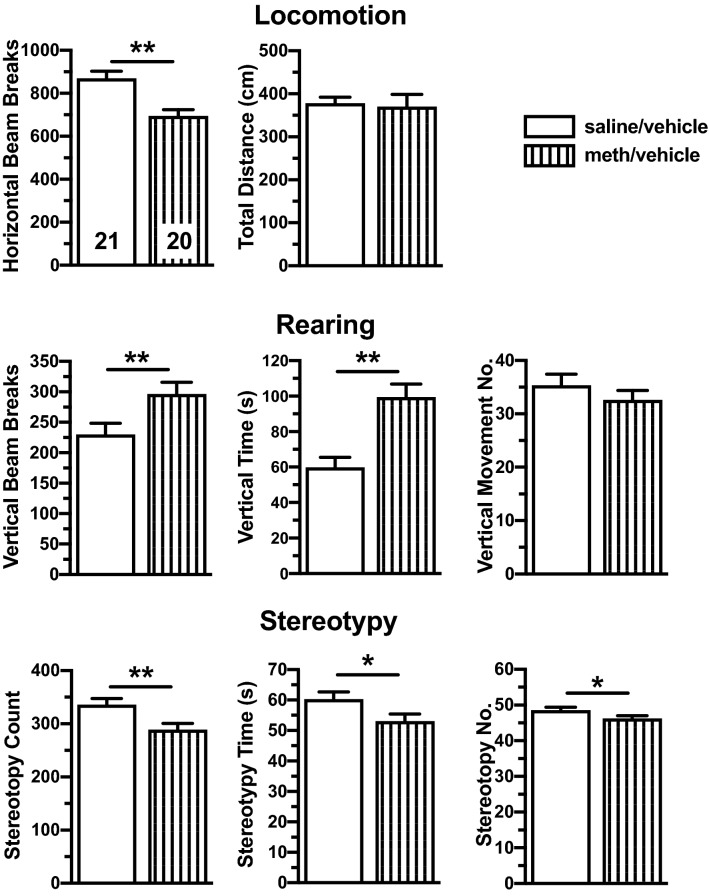
Peak effects of methamphetamine (meth)-induced behavioral sensitization. Vertical beam breaks and time were enhanced in meth/vehicle rats. Horizontal activity and stereotypy measures (count, time, and number) were reduced in meth/vehicle rats. One-tailed Student’s t-test, *p < 0.05, **p < 0.01.

Behavioral measures were pooled for all rats that self-administereed meth and received vehicle in the osmotic pump during forced abstinence (meth/vehicle) and compared to the scores for the pooled saline-yoked rats that received vehicle (saline/vehicle). A representative time course is illustrated in Fig. 4. For statistical analyses, data were collapsed within two phases of the meth-induced effect, onset (20–30 min post acute challenge; Fig. 5) and the peak (30–50 min post acute challenge; Fig. 6). During onset, the meth/vehicle rats exhibited increased rearing behavior (vertical beam breaks t_(40)_ = 3.1, *p* < 0.01; vertical time t_(40)_ = 2.7, *p* < 0.01) and reduced stereotypy count (t_(40)_ = 1.8, *p* < 0.05) compared to saline/vehicle rats (Fig. 5). At peak effect, meth/vehicle rats demonstrated increased rearing behavior (vertical beam breaks t_(166)_ = 1.68, *p* < 0.05; vertical time t_(40)_ = 2.6, *p* < 0.01); stereotypy (stereotypy count t_(40)_ = 2.8, *p* < 0.01; stereotypy time t_(40)_ = 3.2, *p* < 0.01; stereotypy number t_(40)_ = 2.0, *p* < 0.05) and horizontal (t_(40)_ = 3.2, *p* < 0.01) were lower compared to saline/vehicle (Fig. 6). These findings demonstrated that meth self-administration was sufficient to induce behavioral sensitization that could be expressed following 61 days of meth abstinence.

### Effect of rotenone on the expression of sensitization

To verify that the capacity of meth to alter behavior was the same regardless of when vehicle was administered, we qualitatively compared motor outcomes after the acute meth challenge in the saline/vehicle rats among the early (R0), mid (R14), and late (R28) forced abstinence groups for the onset and peak phases (Table 3). Controls were similar across all time points.

**Table 3:** Saline/vehicle rats behavioral outcomes (mean ± SEM).

	R0 (n = 8)	R14 (n = 9)	R28 (n = 4)
**Onset of meth effect**			
Horizontal activity	916 ± 64	904 ± 62	707 ± 26
Total distance	413 ± 28	489 ± 35	493 ± 54
Vertical activity	227 ± 32	199 ± 30	164 ± 35
Vertical time	80 ± 23	52 ± 10	54 ± 16
Vertical movement no	35 ± 4	29 ± 3	29 ± 3
Stereotypy count	340 ± 21	348 ± 30	457 ± 51
Stereotypy time	60 ± 4	60 ± 4	63 ± 6
Stereotypy no	49 ± 2	50 ± 2	49 ± 2
**Peak meth effect**			
Horizontal activity	904 ± 58	859 ± 45	827 ± 95
Total distance	385 ± 21	376 ± 22	369 ± 33
Vertical activity	263 ± 26	216 ± 30	199 ± 42
Vertical time	67 ± 10	57 ± 8	54 ± 13
Vertical movement no	39 ± 3	33 ± 3	34 ± 5
Stereotypy count	311 ± 19	344 ± 19	368 ± 25
Stereotypy time	56 ± 4	62 ± 3	64 ± 6
Stereotypy no	48 ± 1	50 ± 1	47 ± 1

Verification that the controls are similar across all time points. Capacity of meth to alter motor activity is the same regardless of when the animals received the vehicle.

**Figure 7:**
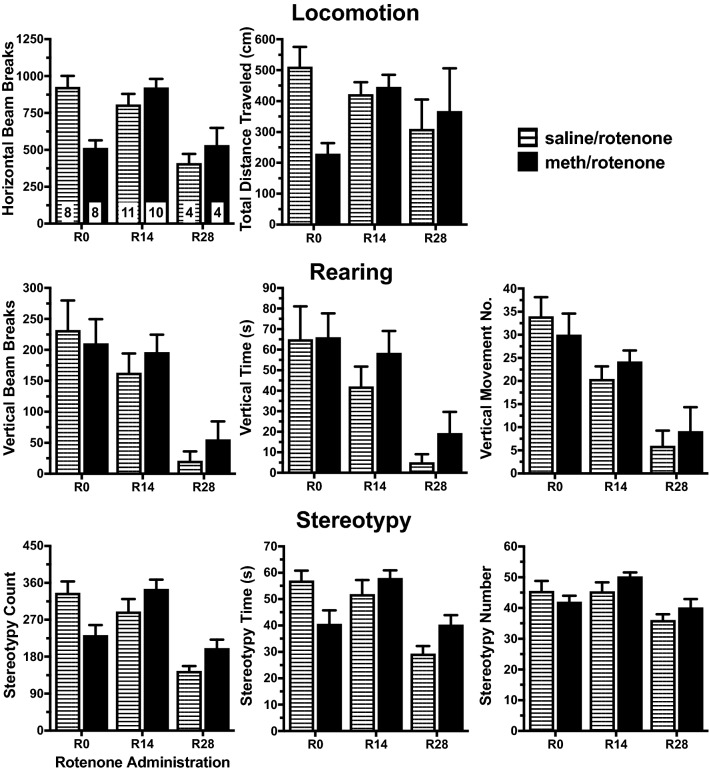
Onset phase motor effects of rotenone administration time on the expression of behavioral sensitization after a methamphetamine (meth) acute challenge. Administration of rotenone to saline-yoked rats on R28 reduced horizontal beam breaks, vertical beam breaks, vertical time, vertical movement number, stereotypy count, and stereotypy time compared to R0 saline/rotenone rats. Compared to R14 saline/rotenone rats, R28 rats had reduced horizontal beam breaks, vertical beam breaks, stereotypy count, and stereotypy number. R14 administration reduced vertical movement number in R14 saline/rotenone rats compared to R0 saline/rotenone rats. R0 meth/rotenone rats had reduced horizontal beam breaks, total distance traveled, and stereotypy count compare to R0 saline/rotenone rats. R14 meth/rotenone rats had increased horizontal beam breaks, total distance traveled, and stereotypy count compared to R0 meth/rotenone rats. R28 meth/rotenone rats had reduced horizontal beam breaks, vertical movement number, and stereotypy count compared to R14 meth/rotenone rats and lower vertical beam breaks, vertical movement number, and stereotypy count compared to R0 meth/rotenone rats. The first bar graph contains the samples size for each treatment group.

**Figure 8:**
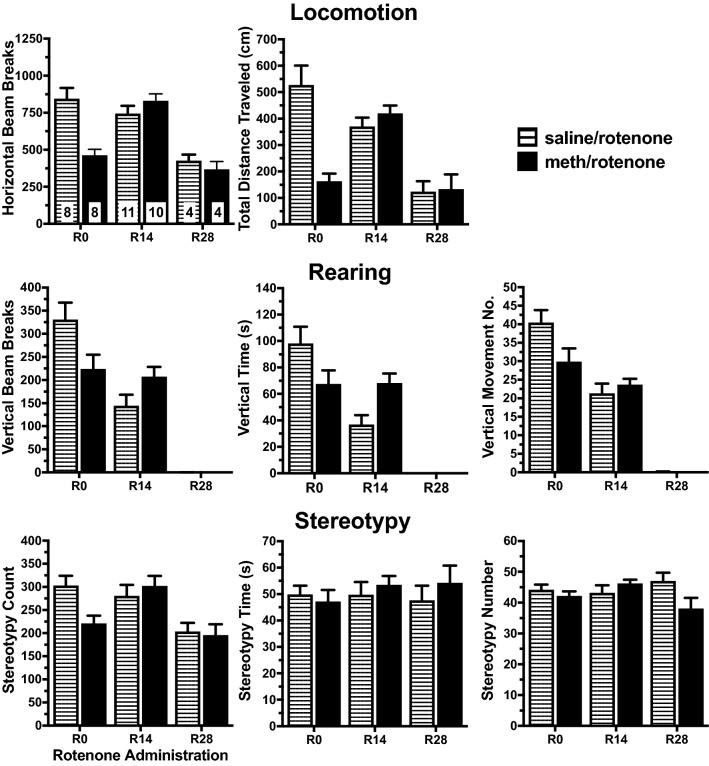
Peak phase motor effects of rotenone administration time on the expression of behavioral sensitization after a methamphetamine (meth) acute challenge. R28 administration of rotenone reduced horizontal bean breaks, total distance traveled, vertical beam breaks, vertical movement number, and vertical time for R28 saline/rotenone rats compared to R0 and R14 saline/rotenone rats. R14 administration reduced in total distance traveled, vertical beam breaks, vertical time, and vertical movement number in R14 saline/rotenone rats compared to R0 saline/rotenone rats. R0 meth/rotenone rats had reduced horizontal beam breaks, total distance traveled, vertical beam breaks, vertical time, and vertical movement number compared to R0 saline/rotenone rats. R14 meth/rotenone rats had increased vertical time compared to R14 saline/rotenone rats. R14 meth/rotenone rats had increased horizontal beam breaks and total distance traveled compared to R0 meth/rotenone rats. R28 meth/rotenone rats had reduced horizontal beam breaks, total distance traveled, vertical beam breaks, vertical time, and vertical movement number compared to R0 and R14 meth/rotenone rats. The first bar graph contains the samples size for each treatment group.

For the following sections on the effect of rotenone on the expression of sensitization, graphical representation of the data can be seen in Fig. 7 (onset phase) and Fig. 8 (peak phase). Statistical anaylses for these data are presented in Table 4 (onset phase) and Table 5 (peak phase). During the onset and peak phases, R28 saline/rotenone group had lower scores for several behaviors, including horizontal beam breaks, vertical beam breaks, vertical time, vertical movement number, stereotypy count, and stereotypy time than both R0 and R14 rats. Compared to R0 saline/rotenone rats, R14 saline/rotenone rats had lower vertical movement number scores during the onset phase. During the peak phase, R14 saline/rotenone rats exhibited reduced total distance measures and all three rearing measures compared to R0 saline rotenone rats. Time-specific post hoc results indicated that the mid (R14) and late (R28) administration of rotenone altered the capacity of the meth acute challenge to elicit a motor response.

**Table 4:** Onset phase motor effects.

Onset phase of responding to acute meth challenge						
	Two-way ANOVA	Between-time Post Hoc within operant treatments			Within-time Post Hoc between operant treatments	
			Saline/rotenone vs saline/rotenone	Meth/rotenone vs meth/rotenone		Saline/rotenone vs meth/rotenone
Horizontal beam breaks	Time: F_(2, 43)_ = 11.8, *p* < 0.01 Treatment: F_(1, 43)_ = 0.9, *p* $\ge$ 0.05 Interaction: F_(2, 43)_ = 9.4, *p* < 0.01	R0 vs R14 R0 vs R28 R14 vs R28	*p* $\ge$ 0.05 *p* < 0.01 *p* < 0.01	*p* < 0.01 *p* $\ge$ 0.05 *p* < 0.01	R0 R14 R28	*p* $<$ 0.01 *p* $\ge$ 0.05 *p* $\ge$ 0.05
Total distance traveled	Time: F_(2, 43)_ = 1.4, *p* < 0.01 Treatment: F_(1, 43)_ = 1.7, *p* $\ge$ 0.05 Interaction: F_(2, 43)_ = 5.2, *p* < 0.01	R0 vs R14 R0 vs R28 R14 vs R28	*p* $\ge$ 0.05 *p* $\ge$ 0.05 *p* $\ge$ 0.05	*p* < 0.05 *p* $\ge$ 0.05 *p* $\ge$ 0.05	R0 R14 R28	*p* $<$ 0.01 *p* $\ge$ 0.05 *p* $\ge$ 0.05
Vertical beam breaks	Time: F_(2, 43)_ = 0.4, *p* $\ge$ 0.05 Treatment: F_(1, 43)_ = 8.9, *p* < 0.01 Interaction: F_(2, 43)_ = 0.4, *p* $\ge$ 0.5	R0 vs R14 R0 vs R28 R14 vs R28	*p* $\ge$ 0.05 *p* < 0.05 *p* < 0.05	*p* $\ge$ 0.05 *p* $\ge$ 0.05 *p* $\ge$ 0.05	R0 R14 R28	*p* $\ge$ 0.05 *p* $\ge$ 0.05 *p* $\ge$ 0.05
Vertical time	Time: F_(2, 43)_ = 6.9, *p* < 0.01 Treatment: F_(1, 43)_ = 0.9, *p* $\ge$ 0.05 Interaction: F_(2, 43)_ = 0.2, *p* $\ge$ 0.05	R0 vs R14 R0 vs R28 R14 vs R28	*p* $\ge$ 0.05 *p* < 0.05 *p* < 0.05	*p* $\ge$ 0.05 *p* $\ge$ 0.05 *p* $\ge$ 0.05	R0 R14 R28	*p* $\ge$ 0.05 *p* $\ge$ 0.05 *p* $\ge$ 0.05
Vertical movement number	Time: F_(2, 43)_ = 16.2, *p* < 0.01 Treatment: F_(1, 43)_ = 0.1, *p* $\ge$ 0.05 Interaction: F_(2, 43)_ = 0.7, *p* $\ge$ 0.05	R0 vs R14 R0 vs R28 R14 vs R28	*p* < 0.05 *p* < 0.01 *p* < 0.05	*p* $\ge$ 0.05 *p* < 0.01 *p* < 0.05	R0 R14 R28	*p* $\ge$ 0.05 *p* $\ge$ 0.05 *p* $\ge$ 0.05
Stereotypy count	Time: F_(2, 43)_ = 10.4, *p* < 0.01 Treatment: F_(1, 43)_ = 0.01, *p* $\ge$ 0.05 Interaction: F_(2, 43)_ = 5.6, *p* < 0.01	R0 vs R14 R0 vs R28 R14 vs R28	*p* $\ge$ 0.05 *p* < 0.01 *p* < 0.05	*p* < 0.05 *p* < 0.05 *p* < 0.05	R0 R14 R28	*p* $<$ 0.05 *p* $\ge$ 0.05 *p* $\ge$ 0.05
stereotypy time	Time: F_(2, 43)_ = 7.4, *p* < 0.01 Treatment: F_(1, 43)_ = 0.004, *p* $\ge$ 0.05 Interaction: F_(2, 43)_ = 4.7, *p* < 0.05	R0 vs R14 R0 vs R28 R14 vs R28	*p* $\ge$ 0.05 *p* < 0.01 *p* < 0.05	*p* $\ge$ 0.05 *p* $\ge$ 0.05 *p* $\ge$ 0.05	R0 R14 R28	*p* $\ge$ 0.05 *p* $\ge$ 0.05 *p* $\ge$ 0.05
Stereotypy number	Time: F_(2, 43)_ = 5.7, *p* < 0.01 Meth: F_(1, 43)_ = 0.6, *p* $\ge$ 0.05 Interaction: F_(2, 43)_ = 1.7, *p* $\ge$ 0.05	R0 vs R14 R0 vs R28 R14 vs R28	*p* $\ge$ 0.05 *p* $\ge$ 0.05 *p* $\ge$ 0.05	*p* $\ge$ 0.05 *p* $\ge$ 0.05 *p* $\ge$ 0.05	R0 R14 R28	*p* $\ge$ 0.05 *p* $\ge$ 0.05 *p* $\ge$ 0.05

Tested variables were rotenone administration time (Time) and operant-task treatment (Treatment). A Newman-Keuls was used for *post-hoc* comparisons.

**Table 5:** Peak phase motor effects.

Peak phase of responding to acute meth challenge						
Two-Way ANOVA		Between-time post hoc within operant treatments			Within-time post hoc between operant treatments	
			Saline/rotenone vs saline/rotenone	Meth/rotenone vs mth/rotenone		Saline/rotenone vs meth/rotenone
Horizontal beam breaks	Time: F_(2, 43)_ = 18.8, *p* < 0.01 Treatment: F_(1, 43)_ = 5.6, *p* $\ge$ 0.05 Interaction: F_(2, 43)_ = 10.6, *p* < 0.01	R0 vs R14 R0 vs R28 R14 vs R28	*p* $\ge$ 0.05 *p* < 0.01 *p* < 0.01	*p* < 0.01 *p* $\ge$ 0.05 *p* < 0.05	R0 R14 R28	*p* $<$ 0.01 *p* $\ge$ 0.05 *p* $\ge$ 0.05
Total distance traveled	Time: F_(2, 43)_ = 13.6, *p* < 0.01 Treatment: F_(1, 43)_ = 6.4, *p* < 0.05 Interaction: F_(2, 43)_ = 13.7, *p* < 0.01	R0 vs R14 R0 vs R28 R14 vs R28	*p* < 0.05 *p* < 0.01 *p* < 0.01	*p* < 0.01 *p* $\ge$ 0.05 *p* < 0.05	R0 R14 R28	*p* $<$ 0.01 *p* $\ge$ 0.05 *p* $\ge$ 0.05
Vertical beam breaks	Time: F_(2, 43)_ = 35.9, *p* < 0.01 Treatment: F_(1, 43)_ = 0.4, *p* $\ge$ 0.05 Interaction: F_(2, 43)_ = 5.7, *p* < 0.01	R0 vs R14 R0 vs R28 R14 vs R28	*p* < 0.01 *p* < 0.01 *p* < 0.01	*p* $\ge$ 0.05 *p* < 0.01 *p* < 0.01	R0 R14 R28	*p* $<$ 0.01 *p* $\ge$ 0.05 *p* $\ge$ 0.05
Vertical time	Time: F_(2, 43)_ = 29.2, *p* < 0.01 Treatment: F_(1, 43)_ = 1.3, *p* $\ge$ 0.05 Interaction: F_(2, 43)_ = 3.1, *p* < 0.05	R0 vs R14 R0 vs R28 R14 vs R28	*p* < 0.01 *p* < 0.01 *p* < 0.01	*p* $\ge$ 0.05 *p* < 0.01 *p* < 0.01	R0 R14 R28	*p* $<$ 0.05 *p* $<$ 0.05 *p* $\ge$ 0.05
Vertical movement number	Time: F_(2, 43)_ = 56.5, *p* < 0.01 Treatment: F_(1, 43)_ = 1.3, *p* $\ge$ 0.05 Interaction: F_(2, 43)_ = 3.4, *p* < 0.05	R0 vs R14 R0 vs R28 R14 vs R28	*p* < 0.01 *p* < 0.01 *p* < 0.01	*p* $\ge$ 0.05 *p* < 0.01 *p* < 0.01	R0 R14 R28	*p* $<$ 0.01 *p* $\ge$ 0.05 *p* $\ge$ 0.05
Stereotypy count	Time: F_(2, 43)_ = 6.2, *p* < 0.01 Treatment: F_(1, 43)_ = 1.3, *p* $\ge$ 0.05 Interaction: F_(2, 43)_ = 3.4, *p* < 0.05	R0 vs R14 R0 vs R28 R14 vs R28	*p* $\ge$ 0.05 *p* $\ge$ 0.05 *p* $\ge$ 0.05	*p* $\ge$ 0.05 *p* $\ge$ 0.05 *p* $\ge$ 0.05	R0 R14 R28	*p* $<$ 0.05 *p* $\ge$ 0.05 *p* $\ge$ 0.05
Stereotypy time	Time: F_(2, 43)_ = 0.3, *p* > 0.05 Treatment: F_(1, 43)_ = 0.4, *p* $\ge$ 0.05 Interaction: F_(2, 43)_ = 0.5, *p* > 0.05	R0 vs R14 R0 vs R28 R14 vs R28	*p* $\ge$ 0.05 *p* $\ge$ 0.05 *p* $\ge$ 0.05	*p* $\ge$ 0.05 *p* $\ge$ 0.05 *p* $\ge$ 0.05	R0 R14 R28	*p* $\ge$ 0.05 *p* $\ge$ 0.05 *p* $\ge$ 0.05
Stereotypy number	Time: F_(2, 43)_ = 0.5, *p* $\ge$ 0.05 Treatment: F_(1, 43)_ = 2.0, *p* $\ge$ 0.05 Interaction: F_(2, 43)_ = 3.3, *p* $\ge$ 0.05	R0 vs R14 R0 vs R28 R14 vs R28	*p* $\ge$ 0.05 *p* $\ge$ 0.05 *p* $\ge$ 0.05	*p* $\ge$ 0.05 *p* $\ge$ 0.05 *p* $\ge$ 0.05	R0 R14 R28	*p* $\ge$ 0.05 *p* $\ge$ 0.05 *p* $\ge$ 0.05

Tested variables were rotenone administration time (Time) and operant-task treatment (Treatment). A Newman-Keuls was used for *post-hoc* comparisons.

Similarly, we compared among the forced abstinence groups in the meth/rotenone rats to determine if the time of rotenone administration had similar effect on capacity of the meth acute challenge to elicit a motor response (Figs. 7 and 8; Tables 4 and 5). R14 rats exhibited lower horizontal beam breaks, total distance, and stereotypy count compared to R0. During the onset phase, R28 rats exhibited lower stereotypy count and time scores and lower horizontal activity and rearing scores compared to R0 and R14 rats; these differences did not persist to the peak phase. All R28 rotenone-treated rats experienced an adverse reaction to the meth acute challenge that resulted in cataleptic-like behaviors soon after the motor test, followed by brief, generalized seizures and death; the meth/rotenone rats died within a few hours after the acute challenge while the saline/rotenone rats died several hours later. Similar to meth-naïve rats, the administration of rotenone in rats with a history of meth altered their response to the meth acute challenge and this response varied with the abstinence time frame of the rotenone treatment.

### Inhibitory effect of rotenone on the expression of behavioral sensitization

During the onset phase of responding to the acute challenge, meth/rotenone rats did not exhibit increased rearing compared to their corresponding saline/rotenone rats (Fig. 7 and Table 4). R0 meth/rotenone rats exhibited reduced scores for several behaviors. An interaction between rotenone and meth occurred for horizontal beam breaks, total distance traveled, stereotypy count and stereotypy time (Table 4, Two-Way ANOVA). Post hoc evaluation revealed that R0 meth/rotenone rats had decreased horizontal beam breaks, total distance traveled, and stereotypy count compared to R0 saline/rotenone rats (Table 4, Within-Time Post Hoc Between Operant Treatments).

During the peak phase, meth/rotenone rats had reduced horizontal and vertical motor measures compared to their saline/rotenone counterparts (Fig. 8 and Table 5 Within Time Post Hoc). Rotenone administration at any time point appeared to diminish the meth-induced expression of behavioral sensitization exhibited by meth/vehicle rats. There was a significant rotenone effect for horizontal beam breaks, total distance traveled, vertical beam breaks, vertical time, and vertical movement number (Table 5, Two-Way ANOVA). There was a significant meth effect for horizontal beam breaks and total distance traveled (Table 5 Two-Way ANOVA). There was a significant interaction for horizontal beam breaks, total distance traveled, vertical beam breaks, vertical movement number, and vertical time (Table 5 Two-way ANOVA). Post hoc evaluation revealed that R0 meth/rotenone rats had decreased horizontal and vertical behaviors compared to R0 saline/rotenone rats (Table 5, Within-Time Post Hoc Between Operant Treatments). R14 meth/rotenone rats had increased vertical time compared to R14 saline/rotenone rats. R28 rats exhibited stereotypy but no change in horizontal or vertical motor behaviors during this phase. These data suggest that functioning mitochondria during abstinence are required for expression of meth-induced behavioral sensitization. Unlike meth/vehicle rats, meth/rotenone rats, independent of the rotenone treatment time, did not exhibit the enhanced vertical behaviors during the acute challenge.

### Impact of rotenone on the expression of behavioral sensitization due to contingency

The effect of rotenone exposure on abstinence days 14–20 on behavioral sensitization from two different contingency protocols was considered. This was accomplished by comparing responding to the acute meth challenge between meth self-administration/rotenone rats and meth non-contingent yoked/rotenone rats (Table 6). Rotenone had no impact on the expression of behaviors that showed sensitization in rotenone-free rats (Table 6). However in the peak phase, non-contingent meth/rotenone rats exhibited significantly reduced total distance traveled (Table 6).

**Table 6:** Comparison of the self-administration and non-contingent administration R14 meth/rotenone animals.

	Onset	Peak
Horizontal beam breaks	*p* $\ge$ 0.05, t_(15)_ = 1.55	*p* $\ge$ 0.05, t_(15)_ = 1.68
Total distance traveled	*p* $\ge$ 0.05, t_(15)_ = 1.60	***p* < 0.01, t**_**(15)**_** = 3.03**
Vertical beam breaks	*p* $\ge$ 0.05, t_(15)_ = 0.57	*p* $\ge$ 0.05, t_(15)_ = 1.17
Vertical time	*p* $\ge$ 0.05, t_(15)_ = 0.90	*p* $\ge$ 0.05, t_(15)_ = 0.37
Vertical movement number	*p* $\ge$ 0.05, t_(15)_ = 0.76	*p* $\ge$ 0.05, t_(15)_ = 0.35
Stereotypy count	*p* $\ge$ 0.05, t_(15)_ = 1.08	*p* $\ge$ 0.05, t_(15)_ = 0.64
Stereotypy time	*p* $\ge$ 0.05, t_(15)_ = 0.43	*p* $\ge$ 0.05, t_(15)_ = 0.20
Stereotypy number	*p* $\ge$ 0.05, t_(15)_ = 0.98	*p* $\ge$ 0.05, t_(15)_ = 0.07

Student’s *t* test with Bonferroni $\alpha$ correction of 0.025 for multiple comparisons.

## Discussion

Study outcomes revealed several previously undescribed features of behaviors that are observed after protracted abstinence from self-administered meth. These include the ability of low, self-titrated doses of meth to produce behavioral sensitization, and the time-related role of mitochondrial function in meth-initiated processes that occur after drug termination. Expanding on the latter, rotenone administration disrupted meth-induced events ongoing during forced abstinence days 0–34 so as to prevent the expression of behavioral sensitization on abstinence day 61.

Repeated exposure to stimulants are well known to induce brain and behavioral changes that outlive the initiating drug. Behavioral sensitization is often used to motorically describe such enduring effects in laboratory animals. We previously demonstrated that meth-induced behavioral sensitization was induced using five, once daily non-contingent subcutaneous (sc) injections of 2.5 mg/kg meth^30,44^. With a 1 mg/kg sc meth acute challenge, the expression of sensitized motor behaviors on abstince day 14 included increases in rearing and stereotypic behaviors, and decreases in horizontal locomotion^44^. On abstinence day 60, only enhanced stereotypic behaviors were expressed during the meth acute challenge^44^. Such bimodal profiles result from several factors that influence meth-induced motor sensitization, including dose and treatment duration for induction, abstenence duration, and acute challenge dose. For example, in rats, 2.5 mg/kg of meth more readily evoke stereotypies than do doses in the 1.0 mg/kg range^44^. In keeping with these prior findings, stereotypic behaviors were not sensitized in the current study wherein the meth doses that the rats self-titrated were 0.9–2.0 mg/kg. These behavioral profiles also reflect “competing behaviors” wherein an increase in one, negatively impacts the ability of the rat to engage in others e.g., an enhancement in rearing resulted in less engagement in locomotion and/or stereotypic behaviors (for further discussion, see reference 44). Thus, the current study provided proof-of-concept for the ability of low doses of meth, self-administered over a 3 h session, to induce behavioral sensitization that could be expressed two months after stopping self-administration. Operant, self-administration protocols allow rats to control the amount and timing of drug intake. To our knowledge, the only published self-administration protocol used to assess stimulant-induced behavioral sensitization was with the stimulant cocaine wherein extended access (6 h sessions for 16 or 21 days) which result in high self-administered doses and sensitized horizontal locomotion expressed 30 days after the last cocaine exposure^58^. Here, we revealed that a short-access self-administration protocol (3 h session/day for 14 days) was sufficient for meth to induce brain adaptations that led to the expression of behavioral sensitization at 61 days of abstinence.

Several studies demonstrate behavioral and biochemical differences in drug-induced effects between self-administration and non-contingent treatment protocols^59–63^. In contrast to operant protocols that allow rats to self-titrate the dose of drug they administer over the study session, non-contingent protocols do not capture motivational aspects of drug-taking that are captured by self-administration^45^. We have previously demonstrated non-contingent administration of meth similar doses to what rats are seen to self-titrate produce persistent rearing and stereotypy sensitized behaviors^30,44^. Here we reveal that meth self-administration leads to the expression of sensitized rearing behaviors. We tested if rotenone impacts sensitized behaviors imposed by both drug admisnitation protocols and showed that neither group of rats expressed behavioral sensitization.

The expression of behavioral sensitization after repeated drug exposure is a behavioral index of drug-induced neuronal plasticity. The molecular processes engaged during neuronal plasticity have high energy requirements that involve mitochondria^3,45,51,64,65^. The presence of behavioral measures of neuronal plasticity after 60 days of abstinence in the current study suggests that such processes were actively occurring during abstinence. Supporting this concept, rotenone exposure during abstinence disrupted subsequent expression of behavioral sensitization, that is, the meth/rotenone treatment groups did not exhibit the enhancement in vertical behaviors seen in meth/vehicle groups. These results suggest that protracted dynamic processes that underpin meth-initiated neuronal plasticity are sensitive to reductions in mitochondrial function. Noteablely, the blunted sensitization occurred using a rotenone treatment protocol that had no motoric effect on its own, and generally without altering motor behaviors that did not exhibit sensitization. That is, at least in the mid (R14) and late (R28) rotenone treatment groups, horizontal and stereotypic behaviors were not diminished. Exceptions occurred for rotenone treatments given during early phases of post-meth abstinence (i.e., R0 treatment group). Here, locomotion, and 2 of 3 measures for stereotypy, were lower in meth/rotenone groups than saline/rotenone. These intriguing observations suggest that the role of mitochondrial function in meth-initiated neural placticity differs temporally for the various behaviors that are influenced by meth.

Although the dose and treatment duration of rotenone was subthreshold to producing motor deficits in the current study, in a parallel biochemical study, we are determining if these treatments can alter mitochondria. Similar to previous work using 2–3 mg/kg/day for 6 days of rotenone treatment^48,49,66^, we measured mitochondrial proteins and complex activity in striatal samples collected from the R14 animals in this study^67^. We demonstrated that 1 mg/kg/day for 6 days rotenone treatment administered on meth abstinence day 14–20 caused cytochrome c translocation in striatal tissues, and reduced activity in some of the mitochondrial electron transport enzymes^67^. These findings indicate that the rotenone treatment used in the current study that was subthreshold to altering motor function, was sufficient to dysregulate mitochondrial function. Thus, supporting the conclusion that mitochondria are critically involved in the expression of meth-induced behavioral sensitization.

Comparisons of responding to the meth acute challenge between saline/rotenone and meth/rotenone groups administered during late abstinence (R28) provided insight into how rotenone history effected a subsequent, singular exposure to meth. All rotenone treated animals exhibited reduced locomotion during the acute challenge behavioral data collection. These animals also exhibited generalized seizures and eventually died. The consequences of toxic rotenone treatments are known to persist beyond clearing the body of the toxin. The current outcomes indicate that even subthreshold treatments of rotenone (i.e., those that are below those that alter motor behavior), produce cellular pathology that persist for weeks and are sufficient to render a normally well-tolerated single dose of meth toxic. As acute meth treatments induce a variety of cellular responses that require energy^3–6^, these outcomes suggest that lingering effects of rotenone also involve mitochondrial dysregulation. It appears that with sufficient time, the mitochondrial damage to low dose rotenone can improve (as what appears to occur in R0 and R14 groups); however, when sufficient time is not achieved, a moderate dose of meth becomes lethal (as what occurred in the R28 group). Administration of rotenone later in the abstinence period may have lowered the capacity of mitochondria to produce energy necessary to buffer detrimental mitochondrial effects of an acute challenge of meth. In the current study, R28 rats exhibited adverse reactions to the acute meth challenge that included seizures. Seizures can result when the brain is unable to meet increased energy requirements imposed by an imbalance of excitatory and inhibitory transmitters^68^. Meth is known to cause a massive efflux of excitatory transmitters. Thus, as only rotenone-treated animals experienced meth-induced seizures, the current results implicate mitochondrial involvement. Further studies are needed to understand the relationship between mitochondrial function and the neuronal process influenced by meth.

In summary, the current study demonstrated the importance of mitochondria in the dynamic processes needed for the expression of behavioral sensitization. Adminitration of the mitochondrial toxin rotenone at various time frames during methamphetamine forced abstinence lead to changes in responsiveness to an acute challenge. The outcomes presented in this study indicate a post methamphetamine time-related role for mitochondria in brain adaptations that underpin long-term behavioral sensitization.
